# Voriconazole‐Associated Periostitis: New Insights into Pathophysiology and Management

**DOI:** 10.1002/jbm4.10557

**Published:** 2021-10-06

**Authors:** Michael J Bennett, Matthew I Balcerek, Edward AD Lewis, Roland LL Zhang, Caroline Bachmeier, Siok Tey, Steven Faux, Laila Girgis, Jerry R Greenfield, Syndia Lazarus

**Affiliations:** ^1^ Department of Endocrinology and Diabetes St Vincent's Hospital Darlinghurst Australia; ^2^ St Vincent's Clinical School, UNSW Medicine Darlinghurst Australia; ^3^ Department of Endocrinology and Diabetes Royal Brisbane and Women's Hospital Herston Australia; ^4^ Department of Rehabilitation Sacred Heart Health Service, St Vincent's Hospital Darlinghurst Australia; ^5^ Heart and Lung Transplant Unit St Vincent's Hospital Darlinghurst Australia; ^6^ Chemical Pathology Pathology Queensland, Royal Brisbane and Women's Hospital Herston Australia; ^7^ Department of Haematology and Bone Marrow Transplantation Royal Brisbane and Women's Hospital Herston Australia; ^8^ Department of Rheumatology St Vincent's Hospital Darlinghurst Australia; ^9^ School of Clinical Medicine – Royal Brisbane Clinical Unit The University of Queensland Herston Australia

**Keywords:** MATRIX MINERALIZATION (BONE MATRIX), OTHER (DISEASES AND DISORDERS OF/RELATED TO BONE), PHARMACOGENOMICS (GENETIC RESEARCH)

## Abstract

Voriconazole‐associated periostitis (VAP) is an underrecognized and unpredictable side effect of long‐term voriconazole therapy. We report two cases of VAP occurring in the post‐transplant setting: a 68‐year‐old lung transplant recipient who required ongoing voriconazole therapy, in whom urinary alkalinization was used to promote fluoride excretion and minimize voriconazole‐related skeletal toxicity, and a 68‐year‐old stem‐cell transplant recipient with a high voriconazole dose requirement, identified on pharmacogenomic testing to be a CYP2C19 ultrarapid metabolizer, the dominant enzyme in voriconazole metabolism. This is the first reported case of pharmacogenomic profiling in VAP and may explain the variability in individual susceptibility to this uncommon adverse effect. Our findings provide new insights into both the management and underlying pathophysiology of VAP. © 2021 The Authors. *JBMR Plus* published by Wiley Periodicals LLC on behalf of American Society for Bone and Mineral Research.

## 1 Introduction

Voriconazole is a frequently used antifungal in the post‐transplant setting and has been associated with periostitis with long‐term use.^(^ ^1^ ^)^ The true prevalence of voriconazole‐associated periostitis (VAP) is unknown; however, retrospective studies suggest this adverse effect may occur in up to 15% of patients on voriconazole.^(^ ^1^, ^2^, ^3^ ^)^ Risk factors for developing VAP are poorly understood, and treatment options are limited. VAP shares common clinical features with subacute fluoride intoxication, including generalized bone pain, raised alkaline phosphatase (ALP), diffuse periostitis/exostoses on X‐ray, and multifocal uptake on radionuclide bone scintigraphy.^(^ ^2^, ^4^, ^5^, ^6^, ^7^ ^)^

Each voriconazole molecule contains three fluorine atoms and 5% of the dose is metabolized to free fluoride.^(^ ^5^ ^)^ Demonstration of elevated plasma fluoride concentration is essential when diagnosing VAP.^(^ ^2^ ^)^ Fluoride ions have a similar size and electrical charge as hydroxide ions but greater affinity for calcium, resulting in fluorapatite replacing hydroxyapatite in the bone matrix, stimulating excess bone formation.^(^ ^8^, ^9^ ^)^ Voriconazole may also directly induce fluoride‐independent osteoblast proliferation.^(^ ^10^ ^)^ No association between serum voriconazole concentration and plasma fluoride level has been observed.^(^ ^1^ ^)^

Discontinuation of voriconazole rapidly normalizes plasma fluoride concentration and resolves clinical and radiological features;^(^ ^2^, ^5^, ^6^ ^)^ however, cessation is not always feasible. Regardless of treatment course, factors underlying individual susceptibility to this seemingly unpredictable and severe adverse drug effect are poorly understood. Herein, we report two cases with unique insights into VAP pathophysiology and management.

## 2 Case Report

### 2.1 Patient 1

A 68‐year‐old woman presented with a 12‐month history of myalgias and weight loss and a 4‐month history of progressive lower limb weakness, lethargy, and pain affecting her shoulders, hips, elbows, and hands. Her symptoms had become so severe that she required regular long‐acting opioids and assistance with mobility and personal care. She had received a left lung transplant 18 months earlier for pulmonary fibrosis and emphysema. Transplantation was complicated by *Lomentospora prolificans* colonization with mycetoma formation in the right lung, requiring long‐term treatment with voriconazole and terbinafine.

Osteoporosis without previous fragility fracture was diagnosed 3 years earlier (femoral neck *T*‐score −3.0 SD), treated with denosumab 60 mg subcutaneously 6 monthly. She had well‐controlled type 2 diabetes and primary hypothyroidism.

Her regular medications included immunosuppressants (tacrolimus, mycophenolate, prednisone), antimicrobials (azithromycin, trimethoprim/sulfamethoxazole, valganciclovir, terbinafine, oral voriconazole 400 mg three times daily), colecalciferol, levothyroxine, and metformin.

Her weight was 58.7 kg (body mass index [BMI] 21.8 kg/m^2^). She had proximal myopathy and tender, bony nodules on the proximal radius and several phalanges without digital clubbing.

Initial investigations (Table 1) {TBL 1} revealed hypophosphatemia, secondary hyperparathyroidism, and normal 25‐hydroxyvitamin D, renal function, and magnesium. Despite treatment with denosumab, her bone turnover markers (C‐terminal telopeptide of type 1 collagen [CTX] and procollagen type 1 N propeptide [P1NP]) were inappropriately elevated. Voriconazole trough concentration was supratherapeutic (6.2 mg/L, target 1 to 2 mg/L); however, the target range was achieved after a series of voriconazole dose reductions (1.0 mg/L while on 850 mg/d). Her 24‐hour urine collection demonstrated renal calcium conservation and phosphate wasting, with elevated plasma fibroblast growth factor 23 (FGF‐23).

**Table 1:** Initial (Time of Presentation With Bone Pain) and Subsequent Investigations Performed for Patients 1 and 2

Parameter	Patient 1			Reference range	Patient 2		Reference range
	Initial	1 month	2 months		Initial	2 months	
Biochemistry							
Creatinine	72	74	67	45–90 μmol/L	139	108	64–108 μmol/L
eGFR	74	72	81	>60 mL/min/1.73 m^2^	44	60	>60 mL/min/1.73 m^2^
Corrected calcium	2.30	2.41	2.45	2.10–2.60 mmol/L	2.37	2.31	2.10–2.60 mmol/L
Phosphate	0.58	0.91	0.93	0.70–1.50 mmol/L	1.50	1.13	0.75–1.50 mmol/L
PTH	25.6	7.7	6.2	2.0–9.0 pmol/L	3.1	–	1.6–6.9 pmol/L
25(OH)D	65	–	–	50–150 nmol/L	66	–	50–150 nmol/L
1,25(OH)_2_D	93	–	–	60–200 pmol/L	–	–	60–200 pmol/L
Albumin	28	–	–	33–48 g/L	37	31	35–50 g/L
ALP	107	254	208	30–110 U/L	217	99	30–110 U/L
CTX	307	–	–	50–800 ng/L	1480	900	50–800 ng/L
P1NP	104	–	–	8–84 ug/L	132	48	8–84 ug/L
FGF‐23	189.0	–	–	23.3–95.4 ng/L	–	–	–
Voriconazole concentration	6.2	3.4	1.0	3.0–4.0 mg/L	2.4	–	3.0–4.0 mg/L
24‐hour urine analysis							
Urinary calcium excretion	0.6	–	–	2.5–8.0 mmol/d	–	–	NA
Urinary phosphate excretion	30.2	–	–	mmol/d	–	–	NA
Tmp/GFR	0.12	–	–	0.80–1.35 mmol/L	–	–	NA
Urine metabolic screen	Negative	–	–	NA	–	–	NA
Urine glucose concentration	0	–	–	0 mmol/L	–	–	NA
Medications							
Voriconazole dose	1200	900	850	mg/d	800	0	mg/d
Calcium dose	0	500	500	mg/d	600	600	mg/d
Phosphate dose	1000	Nil	0	mg/d	0	0	mg/d
Calcitriol dose	0	0	0.5	mcg/d	0	0	mcg/d
Performance status							
Mobility	Full assist	Standby assist 4WW	Independent 4WW	NA	4WW and wheelchair	Independent	NA
Body weight	58.7	61.0	62.5	Kg	66.7	69.4	Kg

eGFR = estimated glomerular filtration rate; PTH = parathyroid hormone; 1,25(OH)D = 25‐hydroxyvitamin D; 1,25(OH)_2_D = 1,25‐dihydroxyvitamin D; ALP = alkaline phosphatase; GGT = gamma‐glutamyl transferase; AST = aspartate aminotransferase; ALT = alanine aminotransferase; CTX = C‐terminal telopeptide of type 1 collagen; P1NP = procollagen type 1 N propeptide; FGF‐23 = fibroblast growth factor‐23; TmP/GFR = renal tubular reabsorption of phosphate; conc = concentration; 4WW = four‐wheeled walker; NA = not applicable.

No reference range for urinary phosphate excretion is listed as interpretation is relative to other parameters.

**Fig. 1:**
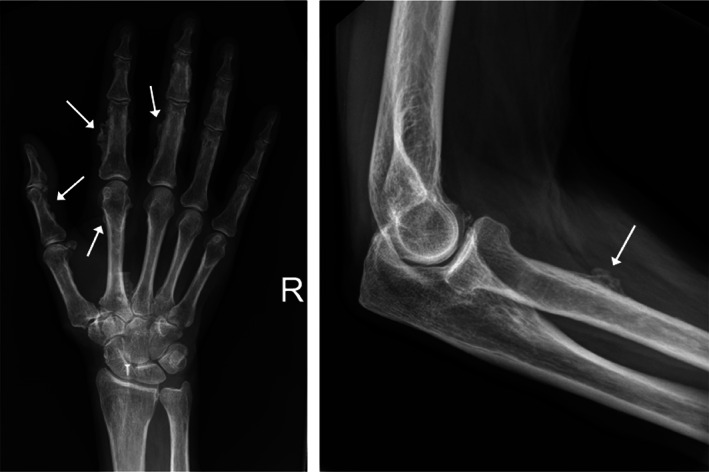
Radiological changes evident 21 months post‐transplant (patient 1). Areas of thick periosteal reaction (arrows) were evident on the middle and proximal phalanges and second metacarpal of the right hand (left image) and right proximal radial shaft (right image).

**Fig. 2:**
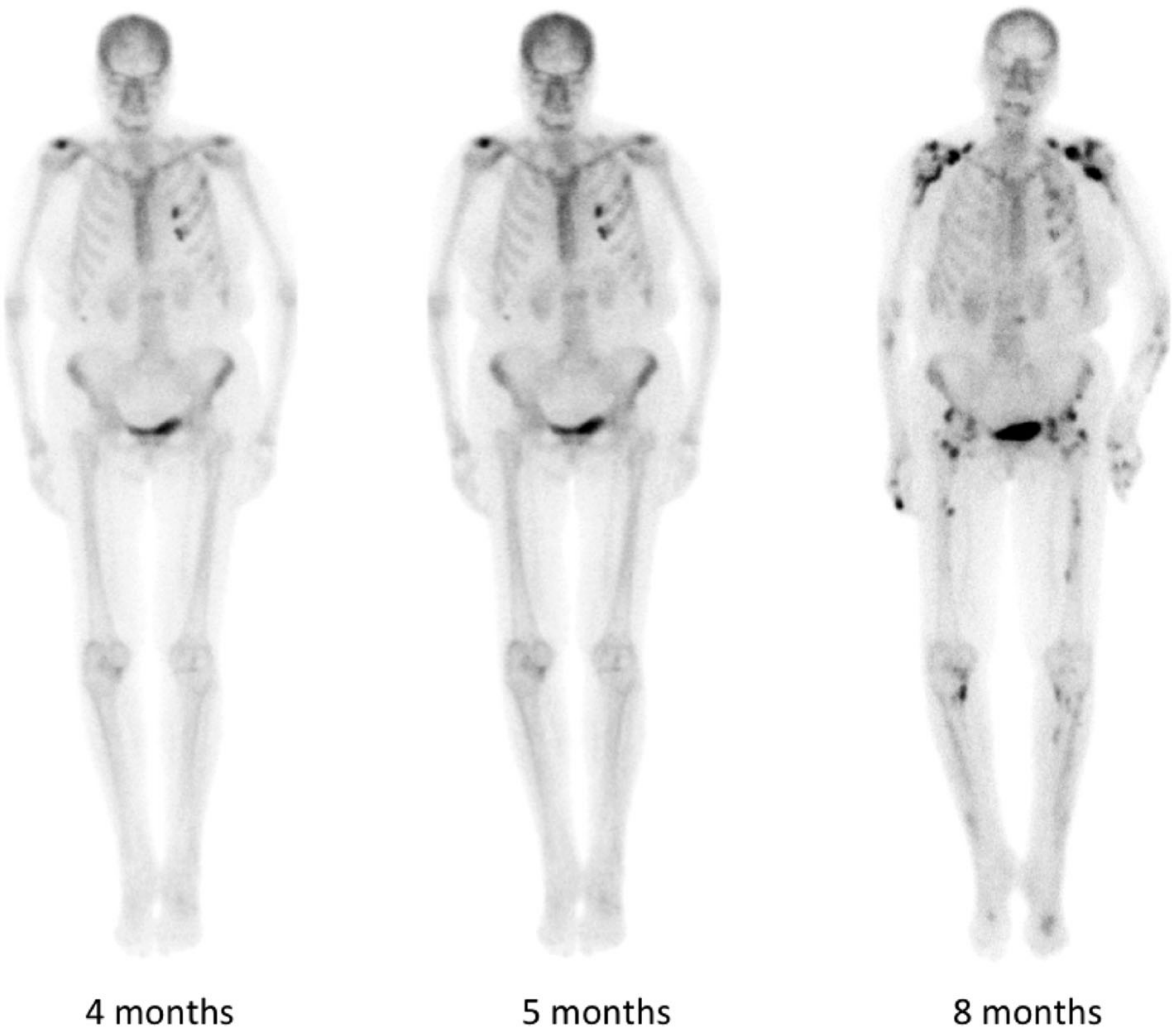
Serial technetium‐labeled bone scintigraphy (patient 1) performed in the months after onset of skeletal pain. Images show the emergence and progression of multiple areas of abnormal tracer uptake affecting the vertebrae (thoracic and lumbar), bilateral shoulders, elbows, pelvis, femora, tibias, hands, knees, and mid‐foot joints. In a number of regions, increased uptake corresponded to areas of periosteal new bone formation.

Skeletal X‐rays showed widespread ill‐defined calcific deposits, consistent with periosteal reaction (Fig. 1). {FIG1} Radionuclide bone scintigraphy demonstrated abnormal tracer uptake corresponding to areas of periosteal bone formation (Fig. 2). {FIG2} FDG‐PET, DOTATATE‐PET, and parathyroid scintigraphy were normal. Dual‐energy X‐ray absorptiometry (DXA) reported normal *T*‐scores in the lumbar spine and osteopenic *T*‐scores in the right femoral neck (−2.0 SD) and total hip (−1.4 SD), an increase in the latter of 25% over 3 years.

VAP was suspected secondary to fluoride toxicity, confirmed by a plasma fluoride concentration of 31 μmol/L (1 to 4 μmol/L). Denosumab was ceased and calcium/calcitriol supplementation commenced. Unfortunately, bronchoscopy identified a *Lomentospora* isolate sensitive only to a combination of voriconazole and terbinafine.

**Fig. 3:**
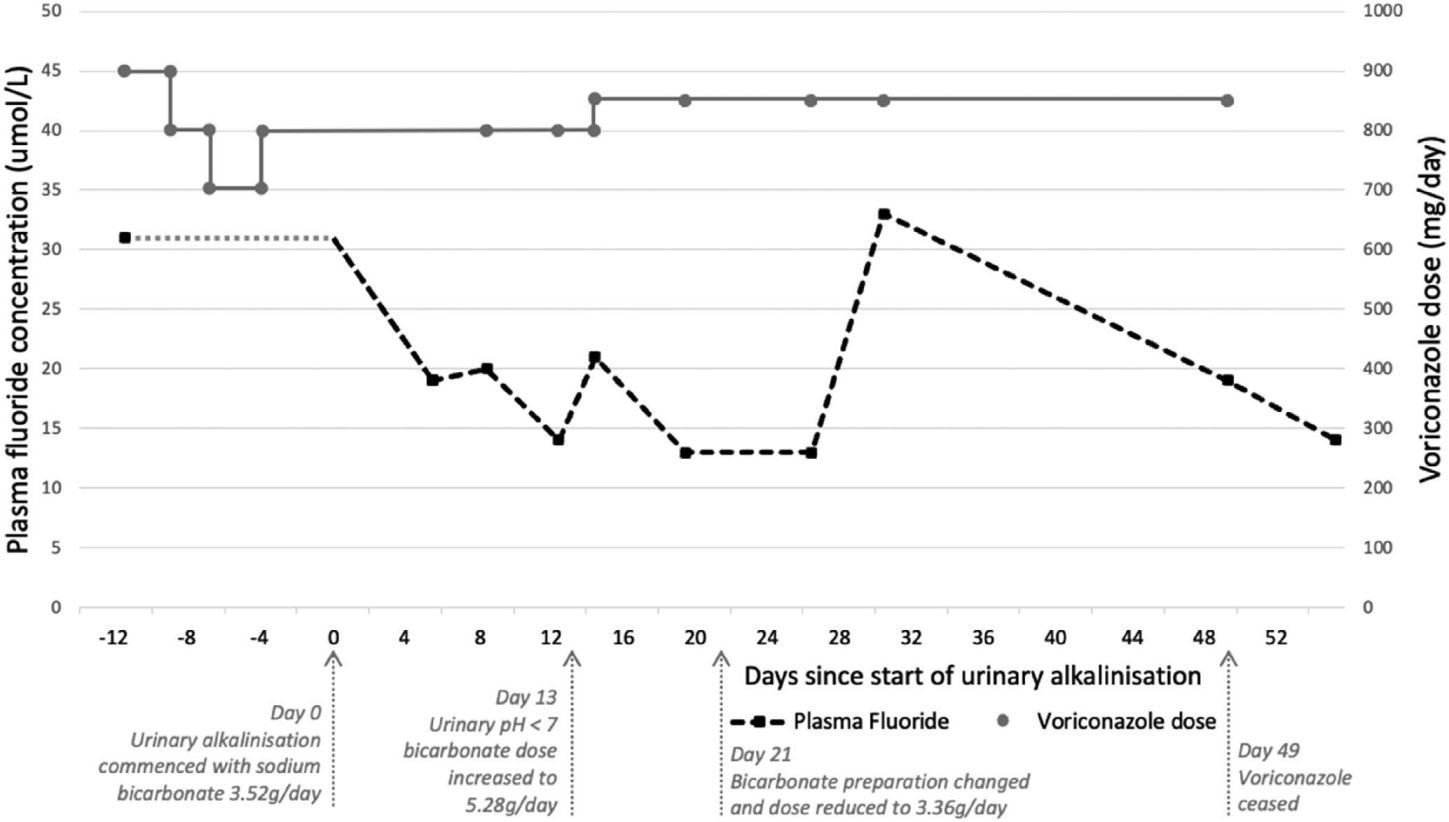
Plasma fluoride concentration and voriconazole dose versus time from commencement of urinary alkalinization for patient 1. Pretreatment plasma fluoride concentration is unknown (represented by the gray dotted line).

Because no alternative antifungal was available, urinary alkalinization with oral sodium bicarbonate 3.52 g/d (given as part of a proprietary solution [Ural, Aspen Pharmacare, Durban, South Africa] containing sodium bicarbonate 1.76 g, tartaric acid 890 mg, citric acid 720 mg, and sodium citrate 630 mg twice daily) was commenced to promote fluoride excretion (Fig. 3). {FIG3} Bedside urine pH was measured daily (target pH >7.5) while in hospital. Her bicarbonate dose was increased on day 13 of alkalinization to 5.28 g/d after a reduction in urine pH. On day 21, due to issues with treatment cost and tolerability and given plasma fluoride had decreased on lower‐dose therapy, treatment was changed to 840 mg sodium bicarbonate capsules given as 3.36 g/d. Plasma fluoride concentration gradually reduced to a nadir of 13 μmol/L on day 26 despite minimal changes to voriconazole dosing. By discharge on day 30, she was able to stand unassisted and mobilize with a four‐wheeled walker.

Plasma fluoride on discharge was at pre‐alkalinization levels (33 μmol/L); however, this result was not available until 2 weeks later, coinciding with deteriorating respiratory function requiring readmission on day 51. A decision was made to withdraw voriconazole treatment on day 51 and bicarbonate treatment on day 55, and she died of respiratory failure 6 days later. Her plasma fluoride at cessation of voriconazole was 19 μmol/L.

### 2.2 Patient 2

A 68‐year‐old White man presented in May 2020 with a 6‐week history of diffuse bone pain, refractory to analgesics. He appeared frail and mobilized with a four‐wheeled walker, a marked deterioration from 3 months earlier when he was independent with mobility and activities of daily living.

His background was significant for high‐risk acute myeloid leukemia, diagnosed 10 months prior. Despite posaconazole prophylaxis, induction and consolidation chemotherapy was complicated by *Aspergillus fumigatus* pneumonia, and he commenced oral voriconazole 200 mg twice daily 7 months before presentation. He subsequently underwent haploidentical stem cell transplant after reduced‐intensity conditioning with melphalan, fludarabine, and 2 Gy total body irradiation. Graft‐versus‐host disease prophylaxis consisted of post‐transplant cyclophosphamide followed by tacrolimus and mycophenolate.

Regular medications at presentation included immunosuppressants (tacrolimus, mycophenolate, low‐dose prednisone), antimicrobials (oral voriconazole 400 mg twice daily, valganciclovir, trimethoprim/sulfamethoxazole), analgesics, and a combination calcium/colecalciferol supplement.

He weighed 66.7 kg (BMI 21 kg/m^2^) and was unable to stand unsupported due to proximal muscle wasting. He could not raise his arms above his head due to pain‐restricted range of motion. No focal bony tenderness was elicited on palpation.

Initial biochemistry demonstrated increased markers of bone turnover (ALP 217 U/L [30 to 110 U/L]) and acute renal impairment (Table 1). Radionuclide bone scintigraphy showed diffuse periarticular radiotracer uptake (Supplemental Fig. S1). Skeletal survey revealed corresponding sites of solid periosteal reaction and soft tissue calcification (Supplemental Fig. S2). DXA reported osteopenic *T*‐scores in the left and right femoral neck (−1.6, −1.9 SD, respectively), with a normal lumbar spine *T*‐score.

**Fig. 4:**
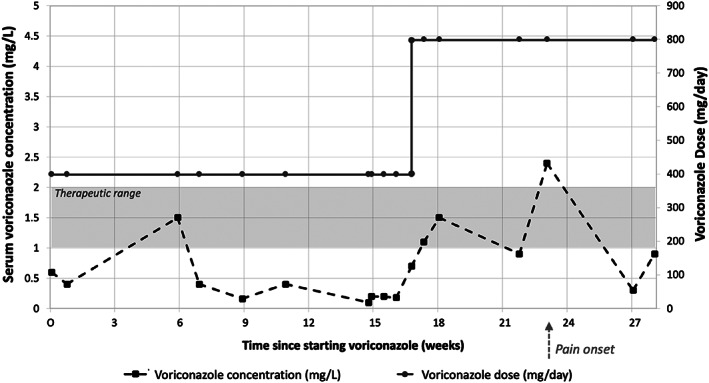
Serum voriconazole concentration from time of commencement of voriconazole for patient 2. Serum voriconazole levels were persistently subtherapeutic and pain onset occurred 6 weeks after dose escalation.

Differential diagnoses included chemoradiation‐related changes and VAP. Notably, 3 months before presentation, oral voriconazole was increased to 400 mg twice daily due to persistently subtherapeutic voriconazole trough levels <1 mg/L, with pain onset 6 weeks later (Fig. 4). {FIG4} VAP was suspected and confirmed by a plasma fluoride level of 26 μmol/L.

Voriconazole was ceased and pain improved within 2 weeks. By 8 weeks, there was remarkable clinical, biochemical, and radiological improvement. His pain completely resolved, and he was mobilizing independently with markedly improved quality of life. Plasma fluoride levels reduced to <10 μmol/L with a concordant decrease in bone turnover markers (Table 1). Repeat bone scintigraphy demonstrated interval decrease in osteoblastic activity.

To investigate the cause of his persistently subtherapeutic voriconazole levels, despite relatively high administered doses, pharmacogenomic studies were performed on stored, pre‐transplant DNA. This identified CYP2C19 ultrarapid metabolizer status (CYP2C19*17/*17), with normal CYP2C9 (*1/*1) and CYP3A4 (*1/*1) metabolism.

## 3 Discussion

**Fig. 5:**
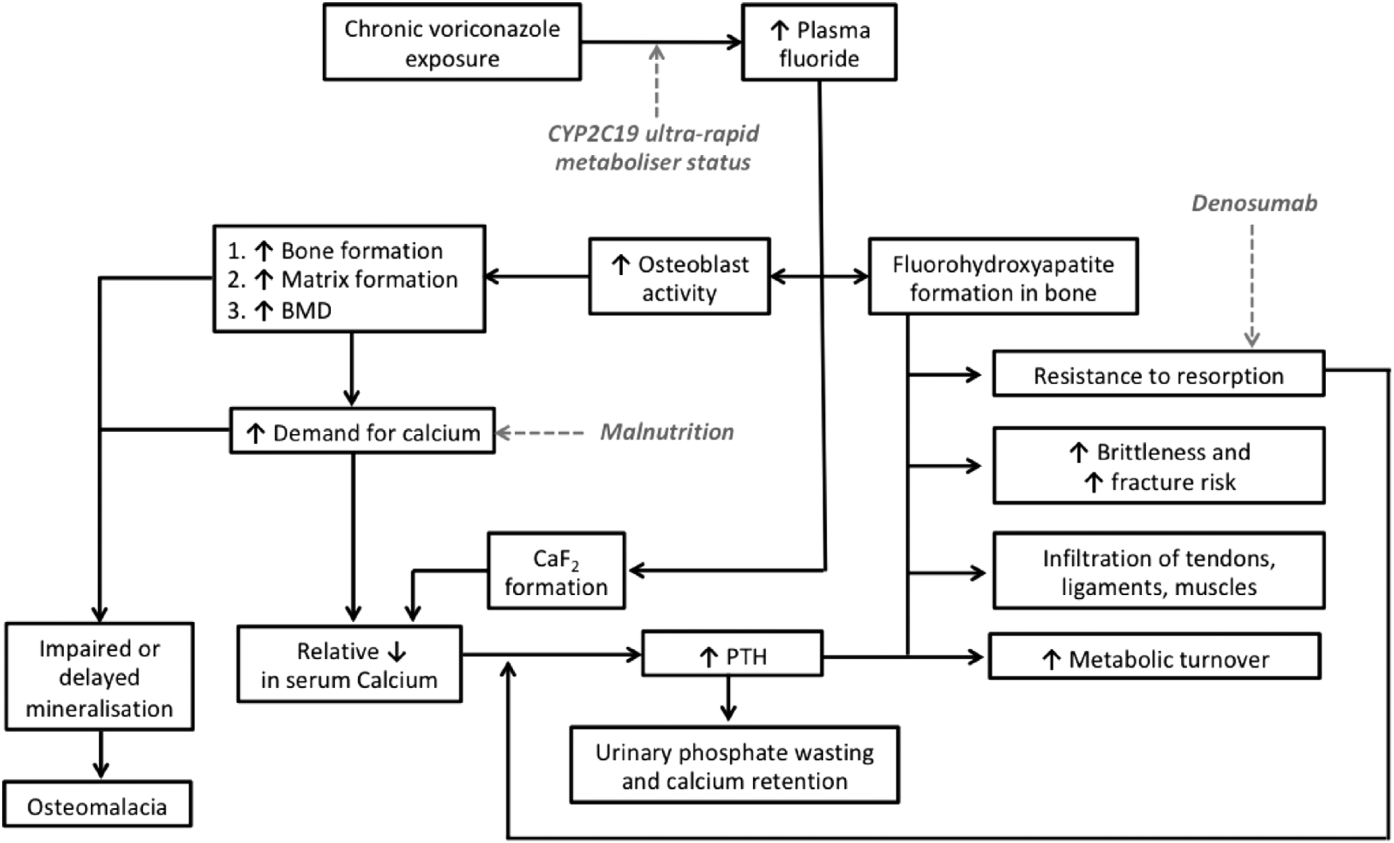
Proposed pathophysiological model incorporating case‐specific factors.

Excess fluoride exposure has deleterious cellular, mineral, and hormonal effects on bone (Fig. 5). {FIG5} Fluoride has anabolic effects, stimulating osteoblasts to make excessive unpermineralized bone (osteomalacia), which accumulates in the periosteal/endosteal regions.^(^ ^11^ ^)^ Deposition of fluorapatite results in denser but brittle bone matrix that is resistant to resorption.^(^ ^9^, ^12^ ^)^

Discontinuing voriconazole is the only curative treatment for VAP, with symptom resolution usually occurring within 2 months post‐cessation.^(^ ^5^ ^)^ Supportive care involves analgesia, correction of malnutrition, and treatment of hyperparathyroidism with calcium/calcitriol. As fluoride absorption, distribution, and excretion are pH‐dependent, an alternative approach may be to decrease plasma fluoride by increasing its elimination through urinary alkalinization. This strategy has been studied in patients undergoing enflurane anesthesia.^(^ ^11^, ^13^ ^)^ After urinary alkalinization, patient 1's plasma fluoride, pain, and mobility improved over a 4‐week period. The long‐term safety and efficacy of bicarbonate therapy in preventing disease progression, however, requires further investigation.

Individuals who develop periostitis have higher plasma fluoride concentrations (12.8 versus 3.6 μmol/L) as well as daily (780 versus 450 mg) and cumulative (130.5 versus 94.7 g) voriconazole doses compared with those without skeletal disease.^(^ ^2^ ^)^ Mean plasma fluoride concentrations >8 μmol/L are associated with skeletal toxicity.^(^ ^2^, ^5^ ^)^ Patient 2's daily voriconazole dose was 800 mg with a cumulative exposure of 138 g. His persistently subtherapeutic voriconazole levels and markedly elevated plasma fluoride concentration led us to hypothesize that rapid metabolism of voriconazole may play a role in the pathogenesis of VAP.

Voriconazole demonstrates nonlinear pharmacokinetics with substantial inter‐ and intraindividual variability.^(^ ^14^ ^)^ Ninety‐eight percent of the drug undergoes oxidative metabolism.^(^ ^15^ ^)^ Between 40% and 49% of the variation in serum concentration is attributed to variations in cytochrome P450 (CYP) enzymes, particularly CYP2C19 for which voriconazole is a major substrate and to a lesser extent CYP2C9/CYP3A4.^(^ ^16^ ^)^ Genetic polymorphisms in the CYP2C19‐encoding gene considerably influence voriconazole dose requirements and subsequent voriconazole exposure, given the variety of metabolizer phenotypes (ultrarapid/extensive/moderate/poor metabolizers).

Consistent with our hypothesis, pharmacogenomic testing identified patient 2 as a CYP2C19 ultrarapid metabolizer (*17/*17), the incidence of which is approximately 4% in Whites.^(^ ^17^ ^)^ This polymorphism explains his persistently subtherapeutic voriconazole levels and may have predisposed him to developing skeletal fluorosis through ultrarapid metabolism to free fluoride. This association between CYP2C19 ultrarapid metabolizer status and VAP, which links gene variation with treatment response, has not previously been reported.

Autoinduction of voriconazole has also been described in cases of low serum concentrations.^(^ ^18^ ^)^ Although not attempted in our patients, the use of CYP2C19 inhibitors including cimetidine may be a potential strategy to maintain or increase voriconazole concentration, while reducing voriconazole daily dose (and associated fluoride metabolite) in patients with skeletal fluorosis.

Similarly, fluoridated antifungal agents (posaconazole and isavuconazole) do not appear to be associated with skeletal fluorosis. Neither agent is significantly metabolized to free fluoride. Posaconazole does not undergo significant CYP enzyme metabolism and is largely excreted unchanged in the feces, whereas isavuconazole is metabolized by CYP3A4/CYP3A5 to inactive metabolites.^(^ ^3^, ^17^, ^18^, ^19^ ^)^

Patient 2 also sustained an acute kidney injury, which may have contributed to fluoride toxicity given fluoride is predominantly renally excreted. Existing data on this subject are conflicting, as a small case series found no association between renal function and plasma fluoride in voriconazole‐treated patients;^(5^ ^)^ however, an inverse correlation has also been reported.^(^ ^3^ ^)^ Previous studies have not grouped patients with renal impairment by metabolizer status, which may explain study heterogeneity. Reanalysis of patients grouped by CYP2C19 genotype may be more revealing.

In summary, we present two cases of post‐transplant VAP. In patient 1, voriconazole could not be ceased and a novel treatment approach of urinary alkalinization was used to reduce plasma fluoride concentration. In patient 2, pharmacogenomic testing revealed CYP2C19 ultrarapid metabolizer status. To our knowledge, this is the first report directly linking pharmacogenomic results with VAP, providing new insights into pathophysiologic mechanisms. Further study in larger cohorts is required, which may have important implications for the adoption of pharmacogenomic testing for pretreatment identification of at‐risk individuals and to guide voriconazole dose selection.

## Disclosures

All authors state that they have no conflicts of interest.

4

### Peer Review

The peer review history for this article is available at https://publons.com/publon/10.1002/jbm4.10557.

## Supporting information


**Supplemental Fig. S1.** Supporting Information

**Supplemental Fig. S2.** Supporting Information
