# Supplementary figures and images for: Voriconazole‐Associated Periostitis: New Insights into Pathophysiology and Management
Source: JBMR Plus. 2021 Oct 6;6(2):e10557. doi: 10.1002/jbm4.10557 (PMC8861987; doi:10.1002/jbm4.10557)

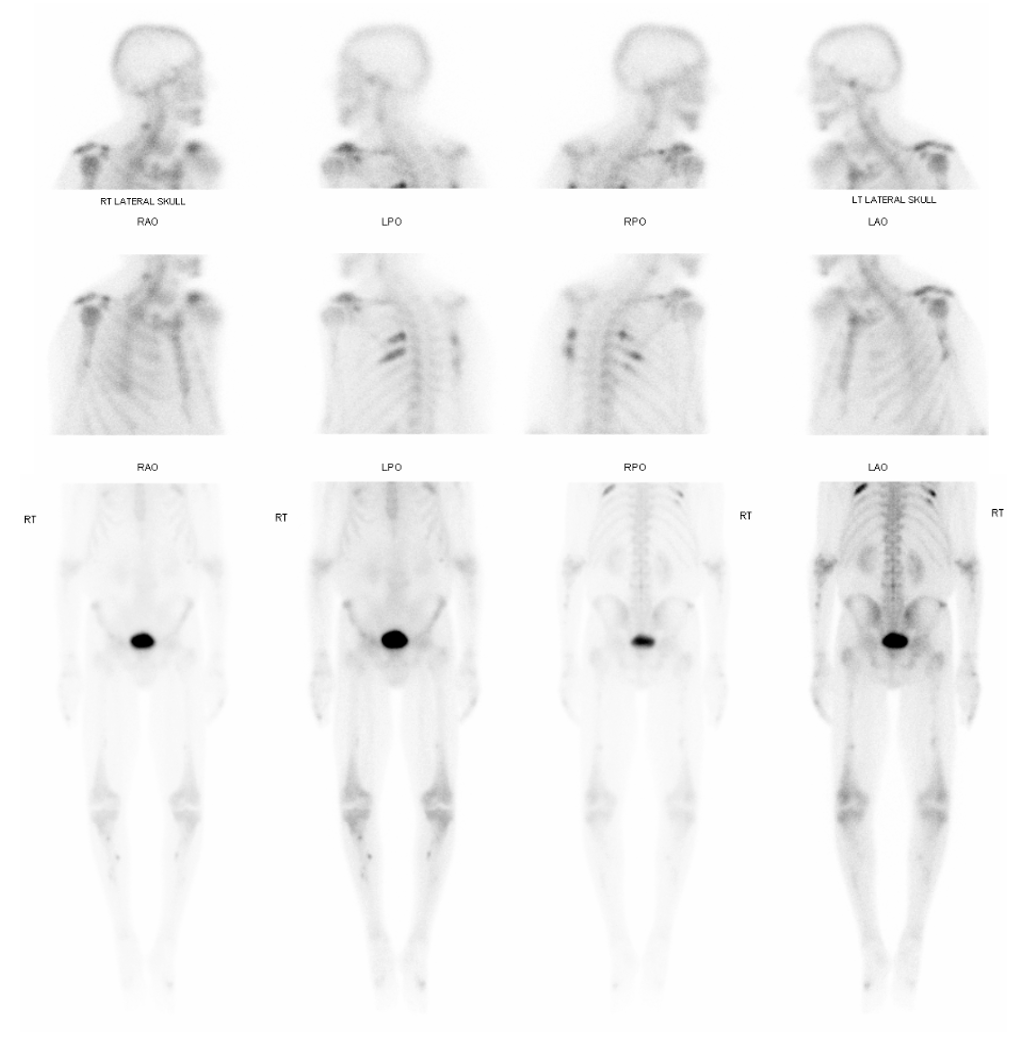

Supplement: Supplementary file 1 — Supplemental Fig. S1. Supporting Information [file JBM4-6-e10557-s002.tiff]

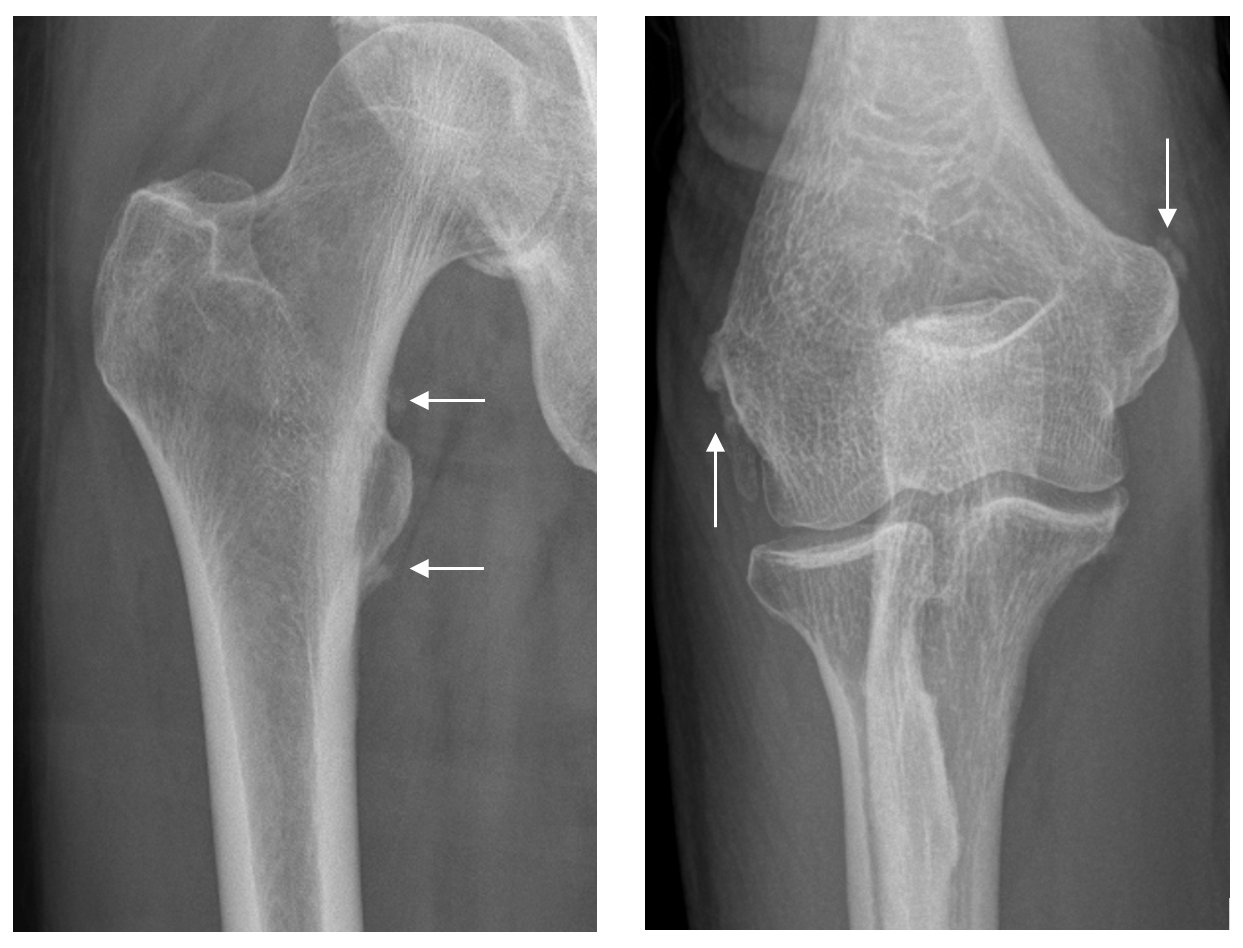

Supplement: Supplementary file 2 — Supplemental Fig. S2. Supporting Information [file JBM4-6-e10557-s001.tiff]
